# Advancing the Field of Pediatric Exercise Oncology: Research and Innovation Needs

**DOI:** 10.3390/curroncol28010061

**Published:** 2021-01-20

**Authors:** Amanda Wurz, Emma McLaughlin, Carolina Chamorro Viña, Sarah L. Grimshaw, Lotta Hamari, Miriam Götte, Sabine Kesting, Francesca Rossi, Patrick van der Torre, Gregory M. T. Guilcher, Krista McIntyre, S. Nicole Culos-Reed

**Affiliations:** 1 Faculty of Kinesiology, University of Calgary, Calgary, AB T2N 1N4, Canada; ajwurz@gmail.com (A.W.); emma.mclaughlin1@ucalgary.ca (E.M.); cchamorro@kidscancercare.ab.ca (C.C.V.); 2 Kids Cancer Care Foundation of Alberta, Calgary, Alberta T2N 1N4, Canada; 3 Murdoch Children’s Research Institute, Melbourne, VIC 3052, Australia; sarah.grimshaw@mcri.edu.au; 4 School of Allied Health, Human Services and Sport, La Trobe University, Melbourne, VIC 3086, Australia; 5 Department of Nursing Science, University of Turku, 20520 Turku, Finland; lotta.hamari@utu.fi; 6 Clinic for Pediatrics III, Department of Pediatric Hematology/Oncology, University Hospital Essen, 45147 Essen, Germany; miriam.goette@uk-essen.de; 7 Department of Sport and Health Sciences, Institute of Preventive Pediatrics, Technical University of Munich (TUM), 80804 Munich, Germany; sabine.kesting@tum.de; 8 Kinderklinik München Schwabing, TUM School of Medicine, Department of Pediatrics and Children’s Cancer Research Center, TUM, 80992 Munich, Germany; 9 Rehabilitation Service, Public Health and Pediatric Sciences Department, A.O.U. Città della Salute e della Scienza—Regina Margherita Children’s Hospital, 10126 Turin, Italy; francesca.rossi@unito.it; 10 Sport and Exercise Center, Princess Máxima Center for Pediatric Oncology, 3584 CS Utrecht, The Netherlands; p.vandertorre@prinsesmaximacentrum.nl; 11 Department of Pediatrics, Cumming School of Medicine, University of Calgary, Calgary, AB T2N 4N1, Canada; greg.guilcher@albertahealthservices.ca; 12 Section of Oncology/Bone Marrow Transplant, Alberta Children’s Hospital, Calgary, AB T3B 6A8, Canada; krista.mcintyre@albertahealthservices.ca; 13 Department of Oncology, Cumming School of Medicine, University of Calgary, Calgary, AB T2N 4N1, Canada; 14 Department of Psychosocial Resources, Tom Baker Cancer Centre, Calgary, AB T2N 4N2, Canada

**Keywords:** childhood cancer, adolescent cancer, patients, survivors, physical activity, implementation

## Abstract

The field of pediatric exercise oncology explores the relationships between physical activity (PA), including exercise, and a range of outcomes among children and adolescents affected by cancer. Although PA is safe and beneficial for this population, several important gaps in knowledge and practice remain. In this article, we describe research and innovation needs that were developed with a team of international experts and relevant literature, a series of online surveys, and an in-person meeting. Addressing these needs will contribute valuable knowledge and practice outputs to advance this field, ultimately enabling a greater number of children and adolescents affected by cancer to realize the benefits of *moving more*.

## 1. Introduction

Pediatric exercise oncology is a field combining medicine, rehabilitation, physiology, kinesiology, and psychology to explore the relationships between physical activity (PA), including exercise, and a range of outcomes among children and adolescents affected by cancer. Observational articles highlight that PA is associated with better physical (e.g., cardiorespiratory capacity), psychosocial (e.g., mood), and cognitive outcomes (e.g., working memory) and that PA may decrease early mortality [1,2]. Experimental articles show that PA interventions can increase PA behaviors (e.g., time spent engaging in PA) and improve physical (e.g., strength), psychosocial (e.g., quality of life), cognitive (e.g., reaction time), and other (e.g., health behavior) outcomes among children and adolescents from cancer diagnosis onward [3,4]. This evidence has been summarized in systematic reviews (e.g., [5]), meta-analyses (e.g., [6]), and, most recently, a comprehensive literature synthesis [7], which collectively suggest that PA is safe and beneficial for children and adolescents affected by cancer.

Nevertheless, advancements in the field of pediatric exercise oncology have been slow, which may be due, in part, to a lack of collaboration and PA guidelines for this cohort. The international Pediatric Oncology Exercise Guidelines (iPOEG) project was undertaken in response. Nine core team members from six countries led the project, identifying and recruiting an additional 122 experts from 21 countries (e.g., clinicians, researchers, and exercise specialists). Key outcomes from this collaborative effort were the iPOEG guidelines and recommendation statements [8] and the iPOEG network. The iPOEG network is comprised of the core team members and additional experts—representing a group of individuals who are dedicated to the study and practice of PA for children and adolescents affected by cancer. An additional outcome, which is the focus of this article, was the identification of research and innovation needs required to advance the field.

To identify the research and innovation needs, the iPOEG network was presented with a list of needs that was drafted based on results from a series of surveys, a literature synthesis, and an in-person meeting, which was attended by article authors [7,8]. Closed-ended questions asked the iPOEG network whether they agreed or disagreed with each need. Consensus was set at ≥80% agreement. As well, open-ended questions collected experts’ comments, input, and prompted whether any needs were missed. The authors reviewed and collated the responses. Table 1 and the text to follow present the collectively agreed upon research and innovation needs.

## 2. Research Needs

Research needs refer to the specific areas of inquiry where further collaborative efforts are required. Overall, more research in the field of pediatric exercise oncology is necessary; however, specific areas necessitate concerted attention (see Table 1). Addressing the following research needs will be critical to advancing knowledge in the field of pediatric exercise oncology.

### 2.1. Collaborating Across Sites to Ensure Adequately Powered Trials

Cancer among children and adolescents is rare [9]. As a result, there are relatively few children and adolescents affected by cancer at any one hospital or clinical site. Underpowered studies hinder researchers’ abilities to draw robust conclusions about the effects of PA. Collaborating, developing, and conducting multisite trials to increase sample size is necessary to enhance statistical power and enable researchers to examine relationships between PA and a range of outcomes. Moreover, adequately powered studies can enable researchers to explore whether PA impacts outcomes for subsets of the population differently (e.g., younger children compared to adolescents or young people affected by blood cancers compared to those affected by bone tumors). Existing networks, such as ActiveOncoKids in Germany, The National Physical Activity and Childhood Cancer Network in Australia (sponsored and run by Little Big Steps), Moving Medicine in the United Kingdom, the Rehabilitation Working Group of the Italian Association of Pediatric Hematology and Oncology in Italy [10], the iPOEG network, and clinical trial registries (e.g., ClinicalTrials.gov), are available to support such efforts.

### 2.2. Conducting Complex Interventions to Successfully Change PA Behavior

Complex interventions are required to successfully change PA behavior among children and adolescents affected by cancer due to the nature of the disease, its treatments, and the many barriers faced by this population [11]. Interventions must consider changing PA behavior (e.g., increasing PA levels by teaching a child how to exercise) and supporting sustainable behavior change (e.g., helping an adolescent move from being sedentary to physically active). Behavior change techniques (BCTs) are methods for changing the psychological determinants of behavior (e.g., one’s confidence to be active) [12]; BCTs are widely viewed as the “active ingredients” underlying PA behavior change interventions. Though BCTs have been used successfully with adults affected by cancer (e.g., goal setting and self-monitoring) [13], few researchers have reported using BCTs with children and adolescents affected by cancer. Michie et al. [14] developed a refined taxonomy of BCTs that may enable researchers in the field of pediatric exercise oncology to incorporate, label, and target BCTs in their research.

Beyond addressing the psychological determinants of behavior, broader models or theories of PA may also be useful. The COM-B model (capability, opportunity, motivation, and behavior) [15] and social ecological models [16] for instance, recognize that behavior occurs within complex systems. Accounting for (and addressing) the multiple and varied influences impacting children’s and adolescents’ PA behavior [17], while considering developmental factors (e.g., dependence, attention span, ability to comprehend instructions, motor control, and language skills) [18], will be critical to increase PA behavior. For example, including parents/guardians (identified as “gatekeepers” to their child’s health) [19] and/or peers [20], depending on the participants age, has been shown to be beneficial for promoting PA. As another example, researchers could consider developing and testing the effectiveness of training educators and exercise specialists in the community to foster safe and autonomy-supportive PA environments and/or developing and testing tools for healthcare providers to facilitate brief conversations about PA with their patients. Such avenues of inquiry may better promote sustainable PA behavior, resulting in positive short- and long-term outcomes.

### 2.3. Identifying Priority Outcomes and Ensuring Consistency in Measurement Tools 

Researchers in the field of pediatric exercise oncology have rarely used patient-oriented approaches and integrated knowledge translation strategies to identify outcomes that are relevant to end users (e.g., healthcare providers, children and adolescents affected by cancer and their parents/guardians, policymakers). Including end users when defining priority outcomes is a necessary first step [21]. Interviews and focus groups are one way to activate the voices of end users; however, researchers could also consider including end user representatives as part of their study team [22].

Connected to identifying priority outcomes is the development of a directory of available, psychometrically sound, and reliable tools (i.e., instruments and techniques used to measure outcomes). To date, a number of different tools have been used to assess outcomes [23], hindering researchers’ ability to compare and pool findings. Favoring tools that have undergone psychometric testing with samples comprised of children and adolescents affected by cancer is recommended. For example, researchers seeking to assess the quality of life could use the Pediatric Quality of Life Inventory [24], whereas those wishing to assess motor performance could use the MOON test [25] or the Gross Motor Function Scale-Acute Lymphoblastic Leukemia [26]. Nevertheless, identifying and using psychometrically sound and reliable tools can be challenging as very few have been tested in this population [5]. For outcomes where such tools are not available, researchers could consider conducting validation studies, collecting qualitative and quantitative data, and/or developing new tools. Having an established directory of psychometrically sound and reliable tools that are important to end users will facilitate impactful research with the potential to be synthesized.

### 2.4. Developing (or Integrating with) Databases (or Registries) to Track PA over Time

Despite the benefits PA can confer, few efforts have been made to develop databases or integrate measures of PA and associated outcomes (e.g., physical, psychosocial, and cognitive outcomes) into existing pediatric oncology databases and registries (see St. Jude Children’s Research Hospital for a notable exception) to explore how these variables change over time. Including measures of PA and associated outcomes may elucidate patterns of change, interactions between variables, and differences within and between subsets of the population over time. Indeed, databases and registries that have included questions about PA have spurred major advancements with regards to the relationships between PA and morbidity and mortality [2]. Including PA and associated outcomes in large databases (or registries) from the point of diagnosis onward will be instrumental for understanding the short- and long-term patterns and impacts of PA, which will provide evidence to better advocate for the role of PA for children and adolescents affected by cancer. Where developing and/or integrating with existing databases (or registries) is not possible, incorporating follow-up measurements within experimental designs can also help researchers study variables over time [27], providing further evidence for the role of PA in the short- and long-term.

### 2.5. Assessing Barriers to PA Implementation

Few researchers have implemented PA for children and adolescents affected by cancer in a way that is sustainable beyond the intervention (or study) period [28]. Among those researchers who have implemented PA, multiple barriers have been described. These include the barriers to PA for children and adolescents affected by cancer (e.g., fatigue, nausea, and deconditioning) [29], as well as the lack of pediatric oncology-specific guidelines, resource constraints, and population considerations [28]. Systematically evaluating and understanding the barriers and facilitators to PA implementation may enable researchers to better plan for sustainability following the intervention (or study) period. The Theoretical Domains Framework [30] and the Consolidated Framework for Implementation Research [31] provide theoretically and empirically informed approaches to investigating constructs that influence implementation effectiveness at multiple levels. These and other frameworks may provide a useful starting point for researchers.

### 2.6. Tailoring to Ensure Safety and Benefits throughout the Cancer Trajectory 

Researchers in the field of pediatric exercise oncology have typically explored the effects of PA among children and adolescents during intensive treatments (e.g., hematopoietic stem cell transplants) [32] or post-treatment [33]. Little has been done at other phases along the cancer trajectory, such as at diagnosis (rehabilitation opportunities), long-term post-treatment (PA maintenance effects), palliation (movement as supportive care), or during transitions between phases. As a result, PA “prescriptions” at each phase to ensure safety and maximize benefits are unknown. To determine these “prescriptions” and enable tailoring, studies comparing different PA “prescriptions” are required. The main components of a PA “prescription” are frequency, intensity, type, and time, and each can be varied or manipulated. For example, during intensive treatments, children and adolescents would be expected to experience fatigue and anemia, suppressed immune system functioning, muscular deconditioning, and social and physical isolation. The tailoring of PA to ensure safety may therefore include sterilization and sanitation procedures, as well as modified frequency, intensity, type, and time of PA—such as starting at lower intensities and progressing slowly. Understanding optimal “prescriptions” can also better inform the benefits to be expected. In this same example, the exercise prescription during intensive treatment (a time when large declines in physical and psychosocial functioning are expected) may be engaged in with the goal of mitigating or maintaining functional declines and symptoms, as opposed to realizing improvements.

### 2.7. Adhering to Reporting Checklist Standards to Enhance Quality

As the field of pediatric exercise oncology continues to advance, ensuring research rigor will be imperative to enhance credibility. Guidelines for designing, conducting, and reporting on varied trial designs should be followed (see Equator Network). Moreover, ensuring clear, transparent, and consistent reporting will be important to produce valid, reliable, and impactful research [34]. Researchers are therefore urged to (i) clearly state their hypotheses and/or research questions; (ii) choose appropriate study designs; (iii) offer rationale to support design choices; (iv) describe the sample, recruitment procedures (e.g., randomization procedures), and intervention in enough detail to ensure replication; (v) document and explain losses to follow-up; (vi) use appropriate and validated tools; (vii) consider intention to treat analysis; (viii) report all results (even when no effect is observed); (ix) consider the biases in their own study; and (x) situate study findings within and beyond the field of pediatric exercise oncology. In addition, highlighting what can and cannot feasibly be done within the context of a PA study, such as the blinding of participants and assessors, is important. Articulating and acknowledging the inability to blind participants in future publications is necessary to open conversations around what is (and is not) quality research in the field of pediatric exercise oncology. Finally, to enhance transparency and replicability, researchers can consider using preprint options (e.g., SPORTRxiv) and publishing and/or registering their protocols.

### 2.8. Considering Alternative Study Designs to Lay a Strong Foundation, Enhance External Validity, and Address the Ethics of Withholding PA

Alternative study designs, in addition to traditionally favored definitive randomized controlled trials, are critical in emerging fields. Specifically, small-scale pilot, cohort, and/or case control studies can lay a strong foundation in advance of definitive randomized controlled studies testing causation hypotheses [35]. Moreover, varied study designs, such as pragmatic randomized controlled trials, active controlled trials, preference-based trials, and hybrid implementation–effectiveness trials, can contribute information that can help better understand barriers to implementation and the effects of PA in “real world” settings [36]. Such external validity may be necessary to promote lasting behavior change and positive outcomes on a larger scale. Finally, alternative study designs may address an important, and often overlooked, ethical consideration—that PA is widely recognized as a protective health behavior [37]—with the United Nations declaring access to PA as a basic right for all children and adolescents [38]. Thus, withholding PA for a control group in a definitive randomized controlled trial may need to be reconsidered. Researchers can reflect on the most appropriate study design that ensures all participants have access to PA (e.g., programs, strategies, and recommendations) in a timely manner.

## 3. Innovation Needs

Innovation needs refer to the methods and processes of motivating large-scale policy and practice changes; disseminating and implementing research and clinical findings; and equipping individuals with the knowledge, skills, and confidence needed to change practice. Yet, few changes have been made to the policy, knowledge translation activities typically end with a peer-reviewed publication, and resources to support practice change are elusive. Addressing the following innovation needs will result in outputs that could effectively change the face of care for children and adolescents affected by cancer (see Table 1).

### 3.1. Developing and Partnering with Networks to Support the Field of Pediatric Exercise Oncology

The relative absence of professional pediatric exercise oncology networks has made advancements in research and innovation difficult. Networks can connect like-minded individuals [39] and provide further avenues for knowledge generation, dissemination, and translation [40]. Indeed, the iPOEG network was created to offer individuals working in, or supportive of, the field of pediatric exercise oncology opportunities to connect, share information on programs and resources, and collaborate. Moving forward, it will be important to establish strong working relationships with other networks who may be able to support advancements in pediatric exercise oncology. Examples of other networks working within pediatric exercise oncology are listed above (see **Research Needs**—*Collaborating Across Sites to Ensure Adequately Powered Trials*), whereas more general pediatric oncology networks could include the Pediatric Oncology Group of Ontario, C^17^, and the International Society of Paediatric Oncology. Partnering with existing networks can support growth and avoid duplicating work.

Beyond professional networks, identifying and getting involved with organizations comprised of children and adolescents affected by cancer and their parents/guardians will be important. Examples of such an organization are the Kids Cancer Care Foundation in Canada, Little Big Steps in Australia, and Sylva in Finland. Amplifying the voices of the individuals comprising these organizations will enable priority outcomes to be identified (see **Research Needs**—*Identifying Priority Outcomes and Ensuring Consistency in Measurement Tools*), support implementation efforts, and facilitate concerted and collective advocacy efforts to advance pediatric cancer care through the inclusion of PA.

### 3.2. Thinking of Advocacy as a Shared Responsibility

Although PA promotion is among a top priority globally (e.g., the World Health Organization), PA advocacy efforts have been minimal within pediatric exercise oncology. Advocacy is a process in which “the actions of individuals or groups attempt to bring about social and/or organizational change on behalf of a particular health goal, program, interest, or population” [41]. To be successful, education and advocacy often co-occur. Thus, it will be the responsibility of those engaged in this field to educate end users on the safety and benefit of PA. Position stands are one way to educate end users by presenting evidence in a concise and accessible manner. Drafting a position stand for pediatric exercise oncology will be a necessary first step. There are many examples available through the American College of Sports Medicine that can serve as a starting point. Concurrently, practice changes (e.g., including exercise specialists in clinical care/hospital settings), new policy, and/or resources to support infrastructure requires advocacy directed to key decision- and policymakers. This can be done through contacting local leaders within one’s community or organization, attending community halls, running for and sitting on boards related to pediatric cancer care, and working to collate voices to support PA (see an Alberta-based exercise oncology petition example here). Advocating for pediatric exercise oncology is a shared responsibility—one that anyone involved with, or interested in, this field may undertake.

### 3.3. Agreeing on Training Requirements to Ensure Competent Exercise Specialists

The iPOEG guideline and recommendation statements define an exercise specialist as an individual with specific pediatric oncology exercise knowledge gained through training and/or clinical experience [8]. Exercise specialists are essential to PA delivery that is safe and beneficial. There are a variety of different designations (e.g., exercise physiologist, physical therapist, kinesiologist, and physiatrist) that differ across provinces/states and countries. Regardless of basic training, the iPOEG network agrees that exercise specialists working with children and adolescents affected by cancer should have additional knowledge and skills, including education in pediatric cancer (in order to modify, adapt, and individualize PA). Further, the ability to ensure participants’ autonomy and facilitate behavior change are important to promote physical literacy and support long-term adherence in this cohort [42]. These additional skills and competencies differ from what most exercise specialists would learn in their basic training. To ensure those working in the field of pediatric exercise oncology have comparable skillsets, a competency checklist will be required along with further training opportunities. Examples of “cancer-specific” trainings to equip exercise specialists to work with adults affected by cancer are the Certified Cancer Exercise Training and the Cancer & Exercise Training for Health & Fitness Professionals; these trainings offer a medical overview of cancer, an introduction to cancer and exercise, screening and assessment techniques/tools, insight into psychosocial considerations/health behavior change techniques, and practical case study examples. Building upon these trainings (and existing introductory pediatric exercise oncology training modules) will be necessary to equip exercise specialists to work with children and adolescents affected by cancer.

### 3.4. Developing Resources and Assessing Readiness to Change Practice

Researchers often disseminate their findings through academic channels (e.g., manuscripts and conference presentations). Though some translate their findings into resources or programs, these efforts are not enough to change behavior or modify practice [43]. Creating resources and programs can spur behavior and practice changes and provide end users with the guidance, knowledge, and tools needed to overcome common barriers to implementation. One example of a project being conducted to develop resources can be seen in work by McLaughlin et al. [44]. This team is conducting a project using a patient-oriented approach to co-create resources (e.g., pamphlets and videos) and a dissemination plan for the iPOEG guideline and recommendation statements with key end users (i.e., children and adolescents affected by cancer, parents/guardians, healthcare providers, exercise specialists, and community-based organizations) [44]. However, beyond developing resources and programs, it is critical to acknowledge end users’ willingness to use the resources/programs and change their behavior and/or practice. One useful tool to assess readiness for change is the Organizational Readiness to Change Assessment instrument [45]. Assessing and responding appropriately to a readiness for change will be important to inform successful and sustainable implementation practices [46].

### 3.5. Shortening the Distance from Research to Practice

It has been estimated that it takes 17 years for research results to effectively change practice [47]. This 17-year gap is problematic, particularly in the field of pediatric exercise oncology, as those individuals diagnosed with cancer as a child or adolescent may age out of the system before they can realize the benefits from the research that is taking place. Learning about knowledge translation through workshops (e.g., Knowledge Translation Canada) is a useful starting place. However, to truly shorten the distance between knowledge and practice, knowledge translation must be viewed as a collective undertaking. Researchers cannot expect healthcare providers, organizations, and/or programmers to “pick up” their successful interventions, just as healthcare providers, organizations, and/or programmers cannot expect researchers to run programs/deliver resources. Sharing the “job” can reduce the burden on any one group. The long-term implementation and maintenance of PA programs can occur by working together—end users and researchers—to plan for sustainability from the start [48]. The Pediatric cancer patients and survivors Engaging in Exercise for Recovery program is one such example where researchers, exercise specialists, and a community-based organization partnered-up to ensure children and adolescents affected by cancer have access to PA [49]. Another example is the ActiveOncoKids network in Germany, wherein a centralized team provides PA counselling and triaging to individuals with specialized expertise (e.g., adventure therapy, physical education, rehabilitation, and motivational counseling) for children and adolescents affected by cancer. Fostering such partnerships and testing different models to promote PA may begin to decrease the distance between knowledge and practice.

## 4. Conclusions

The field of pediatric exercise oncology is in an exciting and emerging state. Although the extant literature suggests PA is safe and beneficial, rigorous foundational efforts alongside more experimental and observational studies are necessary to understand the effects of PA and its relationships with priority outcomes. When innovating and translating this research into practice, mutually beneficial and productive collaborative relationships favoring advocacy and shared responsibility are required. From the perspective of international experts, the research and innovation needs described herein are critical to advance knowledge and spur changes to practice, thereby ensuring that a greater number of children and adolescents affected by cancer experience the benefits of *moving more*.

## 5. Human Rights

All procedures performed involving human participants were in accordance with the ethical standards of the institutional and/or national research committee and with the 1964 Helsinki declaration and its later amendments or comparable ethical standards. Ethical approval for the design and conduct of the larger project was provided by the Health Ethics Board of Alberta—Cancer Care Committee (HREBA.CC-18-0565).

## Figures and Tables

**Table 1:** Research and innovation needs.

Research Needs	Innovation Needs
Collaborating Across Sites to Ensure Adequately Powered Trials	Developing and Partnering with Networks to Support the Field of Pediatric Exercise Oncology
Conducting Complex Interventions to Successfully Change PA Behavior	Thinking of Advocacy as a Shared Responsibility
Identifying Priority Outcomes and Ensuring Consistency in Measurement Tools	Agreeing on Training Requirements to Ensure Competent Exercise Specialists
Developing (or Integrating with) Databases (or Registries) to Track PA Over Time	Developing Resources and Assessing Readiness to Change Practice
Assessing Barriers to PA Implementation	Shortening the Distance from Research to Practice
Tailoring to Ensure Safety and Benefits Throughout the Cancer Trajectory	
Adhering to Reporting Checklist Standards to Enhance Quality	
Considering Alternative Study Designs to Lay a Strong Foundation, Enhance External Validity, and Address the Ethics of Withholding PA	

Notes: Lists are not presented to indicate the order of importance. PA = physical activity.
